# Polymyxin B-direct hemoperfusion therapy could contribute to hemodynamics and outcomes in emergency surgical patients

**DOI:** 10.1186/cc10989

**Published:** 2012-03-20

**Authors:** M Yokota, T Goto, T Harada, M Takeda, R Moroi, M Namiki, A Yaguchi

**Affiliations:** 1 Tokyo Women's Medical University, Tokyo, Japan

## Introduction

Polymyxin B-direct hemoperfusion (PMX-DHP) (Toraymyxin^®^; Toray Medical Co., Tokyo, Japan) has been approved to treat patients with endotoxemia and/or severe sepsis due to Gramnegative infection since 1994 in Japan. However, its efficacy and indication are still controversial. Recently, randomized controlled studies were performed in other countries. Our hypothesis is that PMX-DHP may be useful for emergency-operated patients to eliminate endotoxins from the systemic circulation after removal of the source of infection.

## Methods

From July 1994 to May 2011, all adult patients treated with PMX-DHP in our ICU were included in this retrospective observational study. Patients' clinical and microbiological data were collected from medical archives. The emergency postoperation patients and the medical patients were compared for severity, mortality, and hemodynamic status. Values are expressed as mean ± SD. Data were analyzed by Mann-Whitney U test, chi-square test and Fisher's exact probability test. *P *< 0.05 was considered statistically significant.

## Results

One hundred and sixty-six patients (98 men, 68 women; age range 24 to 92 years (mean 64.7 ± 13.3)) were studied. The mortality rate was 34.9% at 28 days after PMX-DHP. There were 129 (77.7%) emergency surgical patients and 37 (22.3%) medical patients. The APACHE II score on the day of PMX-DHP was not significantly different between surgical and medical patients (20.3 ± 7.0 vs. 19.2 ± 8.1, *P *= 0.417). Mean arterial pressure (MAP) significantly improved in emergency surgical patients before and after PMX-DHP therapy (73.7 ± 24.8 vs. 79.7 ± 26.0 mmHg, *P *= 0.017), while MAP was not statistically different in medical patients (69.7 ± 24.2 vs. 76.7 ± 27.1 mmHg, *P *= 0.178). The inotropic score had no statistical difference between before and after PMX-DHP in both surgical and medical patients (13.2 ± 19.8 vs. 12.6 ± 19.2, *P *= 0.61; 16.8 ± 27.3 vs. 13.8 ± 23.6, *P *= 0.65, respectively). The mortality rates at 28 days, 90 days, 0.5 year and 1 year after PMX-DHP were significantly different between surgical and medical patients (28.7 vs. 56.8, 43.8 vs. 83.3, 52.2 vs. 85.7, 54.5 vs. 91.2%, *P *< 0.0001, respectively).

## Conclusion

MAP increased in surgical patients but did not change in medical patients after PMX-DHP, and the inotropic score was not significantly different in both sets of patients. The mortality was significantly lower in surgical patients than in medical patients.

